# Calcium Channel Blocker Use and the Risk for Breast Cancer: A Population-Based Nested Case-Control Study

**DOI:** 10.3390/cancers14092344

**Published:** 2022-05-09

**Authors:** Victoria Rotshild, Bruria Hirsh Raccah, Muna Gazawe, Ilan Matok

**Affiliations:** 1 Institute for Drug Research, School of Pharmacy, Faculty of Medicine, The Hebrew University of Jerusalem, Jerusalem 9112002, Israel; viki.rotshild@mail.huji.ac.il (V.R.); bruria.hirsh@mail.huji.ac.il (B.H.R.); muna.gazawe@mail.huji.ac.il (M.G.); 2 The Department of Pharmacy, Clalit Health Services, Jerusalem District, Jerusalem 9112002, Israel; 3 Department of Cardiology, Hadassah University Hospital Ein Karem, Jerusalem 9112002, Israel

**Keywords:** calcium channel blocker, breast cancer, case–control study, epidemiology

## Abstract

**Simple Summary:**

Calcium channel blockers (CCBs) are widely used among hypertension and heart disease patients. These drugs are effective and well-tolerated. Some studies have found that patients who used CCBs have a higher incidence of breast cancer (BCa). However, other studies did not find such an association. We investigate whether exposure to CCBs in patients with hypertension is associated with an increased risk of BCa. From a cohort of patients prescribed their first antihypertensive medication between 2000 and 2016, we detected 4875 BCa cases. For each case, we matched ten patients without BCa (controls). We found no association between CCB users and an increased risk of BCa compared to the use of other antihypertensive medications. There was no increase in risk even with longer exposure to CCBs (above eight years) and high doses. Considering that CCBs are a widely used antihypertensive drug class, our results provide important safety information on a population level, especially for patients with increased BCa risk.

**Abstract:**

We investigated whether long-term exposure to calcium channel blockers (CCBs) is associated with an increased risk of breast cancer (BCa). We designed a nested case–control study based on data from the Clalit electronic database, the largest Israeli Health Services organization. All newly diagnosed breast cancer (BCa) cases were selected from a cohort of patients with hypertension. Ten controls were matched for each BCa case. The odds ratios (ORs) of BCa among CCBs users were calculated using multivariate conditional logistic regression analyses. A total of 4875 patients with newly diagnosed BCa were identified from the cohort with a median follow-up of 5.15 years. The exposure to CCBs was not associated with an increased risk of BCa (OR = 0.98; 95% CI, 0.92–1.04). Additionally, there was no association between long-term exposure to CCBs (above eight years) and increased BCa risk (OR = 0.91; 95% CI, 0.67–1.21). Higher cumulative doses of CCBs were not associated with an elevated risk of BCa (OR = 0.997; 95% CI, 0.962–1.034, calculated per 1000 DDD). Based on this large population-based study, long-term exposure to CCBs was not associated with an increased risk of BCa. Considering that CCBs are widely used medications, our results provide important safety information on a population level, especially for patients with an increased risk of BCa.

## 1. Introduction

Breast cancer (BCa) is the most common cancer among women [1]. More than one million women are diagnosed with breast cancer every year [2]. About 4500 women are diagnosed with BCa each year in Israel, and about 900 die from the disease. Furthermore, one out of eight women in Israel may develop BCa, according to the Israel Cancer Association [3]. 

There is inconsistent evidence regarding the increased risk of BCa among calcium channel blocker (CCB) users. Several studies have related CCBs with an increased risk of BCa. Pahor and colleagues (1996) found that CCBs were associated with an increased risk of BCa during 3.7 years of follow-up (hazard ratio (HR) = 1.72; 95% confidence interval (CI), 1.27–2.34), after adjustment for confounding factors [4]. Furthermore, Fitzpatrick and colleagues (1997) reported an increased risk of BCa with the use of CCBs (HR = 2.57; 95% CI, 1.47–4.49) with a dose-dependent effect (HR = 4.48; 95% CI, 1.58–12.75 for high CCBs doses) [5]. Moreover, Li and colleagues (2013) conducted a large population-based case–control study and found higher risks of ductal BCa (odds ratio (OR) = 2.4; 95% CI, 1.2–4.9) and lobular BCa (OR = 2.6; 95% CI, 1.3–5.3) among postmenopausal women that were treated with CCBs for ten years or more [6]. Similar results were observed in additional observational studies [5,6,7,8,9,10]. It has been hypothesized that cellular apoptosis might be influenced by cell exposure to CCBs [11]. miRNA-524-5p-BRI3-Erk pathways in tumor cells might be involved in CCBs’ carcinogenic potential [12].

However, other observational studies and randomized controlled trials could not confirm the positive association between CCB exposure and BCa incidence [13,14,15,16,17,18,19,20,21,22]. In a prospective cohort study, long-term use of CCBs for ten years or more showed no evidence of increased risk of BCa compared to never users of CCBs (HR 0.88; 95 % CI, 0.58–1.33) [19]. Furthermore, in a population-based cohort study conducted on 273,152 women newly treated with antihypertensive drugs, it was concluded that CCBs were not associated with an increased risk of BCa overall (HR = 0.97; 95% CI, 0.91–1.03) [22]. Moreover, Brasky et al. reported no association between exposure to CCBs and the risk of BCa among 28,561 postmenopausal women (HR = 1.06; 95% CI, 0.94–1.20) [20].

A few meta-analysis studies did not report an association between exposure to CCBs and the risk of BCa [23,24]. However, two other meta-analyses did report an association between long-term exposure to CCBs and the risk of BCa [25,26].

A meta-analysis by Chen et al. did not report an association between overall exposure to CCBs and BCa [23]. However, they did report an association between immediate-release CCB use and an increased risk of BCa (OR =1.88; 95% CI, 1.37–2.60). A meta-analysis by Wright et al. reported no association either between exposure to CCBs and BCa (HR = 0.99 (95% CI, 0.94–1.03)) [24]. Nevertheless, a meta-analysis by Thakur et al. reported an association between CCB use and the risk of BCa (OR =1.14; 95% CI, 1.02–1.27), with a statistically significant association between exposure to CCBs of nine years and above and increased risk of BCa [26]. The most recent meta-analysis published in 2021, based on patient-level data from 33 RCTs, found no increased risk of BCa among CCB users compared to other AHTs (HR = 0.95; 95% CI, 0.79–1.16) [27].

While numerous studies evaluated whether CCBs increase the risk of BCa, the inconsistent results might be partially explained by a short follow-up and the differences in methodology of the studies mentioned above. Therefore, we aim to investigate the association between long-term CCB consumption among the Israeli population and the risk of BCa.

## 2. Materials and Methods

### 2.1. Data Source

Data from the computerized databases of Clalit Health Services (CHS) were used in this study. CHS is the largest medical health organization in Israel. It provides ambulatory medical facilities for more than 52% of the Israeli population; this database represents the general population’s demographic characteristics and socioeconomic status (SES) [28]. 

The CHS database has computerized medical data from 2000 and contains comprehensive electronic, clinical and administrative patient-level records. The clinical, demographic and socio-economic data are extracted from inpatient and outpatient clinics, including hospitalizations, primary care, specialist clinics, pharmacies dispensing information, laboratory results, and diagnostic and imaging services [29].

### 2.2. Study Population

The study cohort includes all male and female patients aged over 55 years, registered in the CHS database between 1 January 2001 and 31 December 2016. Only patients that recently began antihypertensive drugs were included in the study cohort. Antihypertensive drugs were categorized by the Anatomical Therapeutic Chemical (ATC) 4 codes (Table S1) [30]. As most of the hypertensive population are above the age of 55, we limited cohort inclusion to ages 55 years and older, similar to previously published studies [5,6,9,14,31]. We excluded patients with less than one year of registration history in the CHS database. At least two years of follow-up were also required for inclusion criteria to consider cancer latency. The date of a first pharmacy supply of an antihypertensive drug defined the cohort entry. Patients with a personal history of cancer diagnosed before the cohort entry date were excluded.

### 2.3. Cases and Controls Selection

Following the previously defined cohort formation, a nested case–control analysis was completed. All new BCa diagnoses, based on the International Classification of Diseases (ICD) 9th, were defined as cases [32]. Chronic diseases and malignancies are validated by a systematic methodology in the CHS database. The validity of the diagnoses in the registry has been shown to be high [33,34,35]. The index date was defined by the date of BCa diagnosis. Each case was matched with ten controls by age (within five years), gender, calendar year of cohort entry, and follow-up duration. The controls were allocated to the same index date as the case they were matched with.

### 2.4. Exposure Assessment 

For all cases and controls, exposure to CCB drugs was obtained by identifying all prescriptions dispensed during the study period (from the cohort entry to the index date). Ever CCB use was defined as receiving at least one prescription of CCB drugs (amlodipine, diltiazem, felodipine, lercanidipine, nifedipine, and verapamil) between cohort entry and the index date. CCB drugs were categorized by the ATC 5 codes (Table S2) [30].

### 2.5. Non-Exposures Assessment

Cases and controls dispensed no CCB drugs during the study period.

### 2.6. Potential Confounders

The models were adjusted for universal confounders, variables known to be associated with the risk of BCa, and variables that might influence the choice of antihypertensive therapy, including socioeconomic status (SES), ethnicity, smoking status, hormone replacement therapy use (categorized by ATC 5 codes, Table S3), family history of BCa, and common comorbidities [30]. Comorbidities were classified using the ICD 9th classification. A comorbidity score was constructed in order to adjust for chronic disease burden. We calculated the comorbidity score by summing several chronic diseases from the list for each patient: hyperlipidemia, ischemic heart disease, heart failure, cardiovascular disease, peripheral vascular disease, chronic kidney disease, and chronic lung disease.

Antihypertensive drugs other than CCBs include angiotensin-converting enzyme inhibitors (ACEIs), angiotensin receptor blockers (ARBs), beta-blockers (BBs), thiazide diuretics, and alpha-blockers, categorized by ATC 4 codes (Table S1).

### 2.7. Statistical Analysis

Descriptive statistics of demographic and baseline characteristics of the study population were evaluated using a two-sided χ^2^ test.

To evaluate the association between CCB exposure and the risk of BCa, compared to exposure to antihypertensive drugs other than CCBs, we applied multivariate conditional logistic regression to calculate the odds ratios (ORs) and 95% CI. We adjusted the models for potential confounders (Table S4).

In the primary analysis, we assessed whether exposure to CCBs is associated with an increased risk of BCa compared to non-CCB antihypertensive drugs in patients above 55 years of age diagnosed with hypertension and treated with antihypertensive drugs.

In a secondary analysis, we assessed the duration effect of CCBs on the risk of BCa compared to non-CCB antihypertensive drugs. CCB exposure was calculated by summing the durations of all CCB prescriptions from cohort entry until the index date. Duration of CCB exposure was evaluated as a continuous parameter with predefined categorized groups (less than eight years and eight years or more).

Additionally, we estimated the dose-dependent association between the risk of BCa and the use of CCBs. The defined daily dose (DDD) of each CCB was used to calculate the cumulative dose of different CCBs and a continuous variable per 1000 DDDs (Table S2) [30]. Finally, we identified whether there was an association between Dihydropyridine and Non-dihydropyridine CCBs and the risk of BCa. We also conducted a sensitivity analysis to evaluate whether exposure to CCBs is associated with an increased risk of BCa compared to non-CCB antihypertensive drugs in female patients.

SPSS 21 (IBM SPSS Statistics, IBM) was used to conduct the statistical analyses.

### 2.8. Ethics Approval

The Clalit Health Services (CHS) institutional ethics committee approved the study protocol. We followed the Strengthening the Reporting of Observational Studies in Epidemiology (STROBE) reporting guidelines to report the study results [36].

## 3. Results

**Figure 1:**
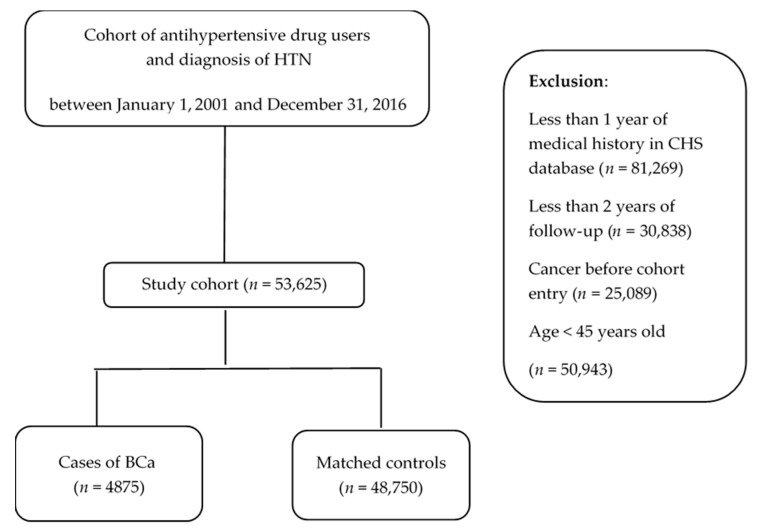
Study flow chart. Following study cohort formation, all BCa cases and matched controls were identified. CHS—Clalit Health Services; HTN—hypertension; BCa—breast cancer.

Following inclusion and exclusion criteria, the study cohort was formed and included 53,625 patients (Figure 1). During the study period, we identified 4875 new cases of BCa; these were matched with 48,750 controls.

Table 1 presents the baseline demographic and clinical characteristics of the cases and matched controls. The mean age at the diagnosis of cases and matched controls was 61.3 years (standard deviation (SD) 9.7 years) with a follow-up of 5.2 years (SD 3.7 years). Most of the study population were ever-used ACEIs/ARBs (72%), half were ever-used BBs (54%), and 40% were ever-used CCBs. The mean duration of ever use of CCBs was 1.96 years (interquartile range 0.08–16.17 years). As expected, cases had a higher rate of family history of BCa (13.2% vs. 5.4% in controls, *p* < 0.01), and a higher proportion of BCa cases were exposed to smoking and hormone replacement therapy (Table 1).

### 3.1. Primary Analysis

**Figure 2:**
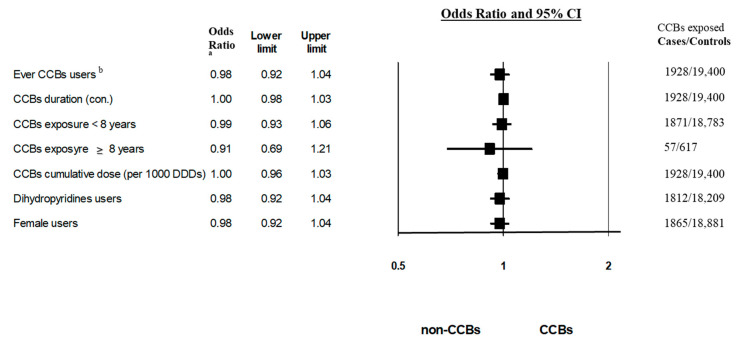
Adjusted odds ratios of breast cancer among calcium channel blocker users. ^a^ Univariate analysis. ^b^ Multivariate analysis, adjusted for socioeconomic status, smoking status, exposure to hormone replacement therapy, family history of breast cancer, and comorbidity score (counting diagnosis of 7 chronic diseases). CCBs—calcium channel blockers; CI—confidence interval; DDD—defined daily dose; OR—odds ratio.

In the primary analysis, ever use of CCBs was not associated with an elevated risk of BCa (adjusted OR = 0.978; 95% CI, 0.918–1.043) when compared to non-CCB antihypertensive drugs in patients over 55 years of age diagnosed with hypertension and treated with antihypertensive drugs (Figure 2). These results were adjusted for socioeconomic status (SES), smoking status, hormone replacement therapy, family history of BCa, and comorbidity score.

### 3.2. Sub-Group Analyses

Our results were consistent over different sub-analyses, including sensitivity analysis.

In a secondary analysis, we did not find an association between the duration of CCB exposure and increased risk of BCa (OR = 1.003; 95% CI, 0.980–1.026 for CCB exposure as a continuous variable) (Figure 2). Moreover, no association was found between long-term exposure to CCBs (over eight years) and increased BCa risk (OR = 0.914; 95% CI, 0.689–1.21) (Figure 2). Our results indicate that higher cumulative doses of CCBs were not associated with an increased risk of BCa (OR = 0.997; 95% CI, 0.962–1.034, calculated per 1000 DDD) (Figure 2). 

More than 88% of CCB users were exposed to Dihydropyridines. Dihydropyridine exposure was not associated with a significant increase in the risk of BCa, based on the results of univariate analysis (adjusted OR = 0.978; 95% CI, 0.918–1.043) (Figure 2). 

### 3.3. Sensitivity Analysis

In the sensitivity analysis, we studied the association between CCB exposure and BCa risk in females. Ever use of CCBs was not associated with a statistically elevated risk of BCa (adjusted OR = 0.989; 95% CI, 0.929–1.053) compared with non-CCB antihypertensive drugs among female patients. This result is comparable with the primary analysis (Figure 2).

## 4. Discussion

Our population-based nested case–control study showed no association between ever use of CCBs and increased risk of BCa (OR = 0.978; 95% CI, 0.918–1.043) compared with non-CCB antihypertensive drugs (Figure 2). Our results were consistent over different sub-analyses, including a sensitivity analysis that included female patients only (OR = 0.978; 95% CI, 0.918–1.042) (Figure 2).

Previously published studies reported duration and dose–response effects of CCB exposure on the risk of BCa [5]. Fitzpatrick et al. reported that high CCB doses were associated with a higher risk of BCa (HR = 4.48; 95% CI, 1.58–12.75) [5]. Li et al. found an increased risk of BCa among postmenopausal women treated with CCBs for over ten years (OR = 2.4; 95% CI, 1.2–4.9 and OR = 2.6; 95% CI, 1.3–5.3, for ductal and lobular BCa, respectively) [6]. To investigate the risk factors for increased risk of BCa among CCB users, we evaluated the duration and dose–response effects of CCB exposure in our cohort of hypertensive patients. Neither higher cumulative doses of CCBs nor long-term exposure to CCBs were associated with an increased risk of BCa (OR = 0.997; 95% CI, 0.962–1.034, calculated per 1000 DDD; and OR = 0.914; 95% CI 0.689–1.21 for CCB exposure above eight years) (Figure 2).

Our study has strengths and limitations. We used a population-based nested case–control design to minimize the selection bias of controls. We also included patients with newly diagnosed hypertension who initiated antihypertensive therapy to diminish confounding by indication.

Moreover, restricting a cohort to patients receiving antihypertensive medications, in contrast to a general CCB-unexposed population, might minimize detection bias, as patients on chronic drug therapy are more likely to be monitored by their clinicians and to go through screening tests, including BCa screening [37].

We evaluated drug exposure based on pharmacy records of medications that were dispensed. Data on medication purchase is an acceptable method to evaluate drug use (duration and dose exposure) in studies using databases [29].

Cases included patients that were newly diagnosed with breast cancer during follow-up. Each case was matched with ten controls by age, sex, calendar year of cohort entry, and duration of follow-up to reduce time trends in the utilization of CCBs and antihypertensive medications incidence throughout the study period. 

Differences were found in some comorbidities among cases and controls. To decrease the potential for confounding bias by health status, the model was adjusted for comorbidity score that included seven common chronic diseases: hyperlipidemia, ischemic heart disease, heart failure, cardiovascular disease, peripheral vascular disease, chronic kidney disease, and chronic lung disease. Moreover, the model was adjusted for socioeconomic status, smoking status, and family history of BCa. The smoking rate evaluated from structured fields of electronic clinical records might be underestimated [29].

In addition, our study has some limitations. A higher smoking rate was seen among cases compared to controls. Thus, the primary analysis was adjusted for smoking status. However, as evaluated from structured fields of electronic clinical records, smoking rates might be underestimated information, as previous studies involving other databases have reported [38].

Our dataset lacks information on grade, stage, and type of BCa cases, so we could not evaluate the association between CCB exposure and BCa type or grade at diagnosis. Competing risk bias could not be controlled due to a lack of information needed to perform a competing risk analysis. However, we can assume that the mortality rates were not different between the exposure groups, as the study population is based on a homogeneous cohort of patients utilizing antihypertensive medications. This study is based on the local Israeli population, so caution should be taken when generalizing the results of this study to other populations. Finally, as in all epidemiologic studies, there is a potential for residual confounders for factors that were not controlled.

## 5. Conclusions

This large population-based study showed that CCBs were not associated with an increased risk of BCa. Our study provides important additional safety information about CCBs, a widely prescribed first-line antihypertensive therapy. This information is essential, especially for subjects with baseline risk factors for BCa (such as BRCA mutation carriers), in reporting no increased risk for BCa following long-term exposure to CCBs.

## Figures and Tables

**Table 1:** Baseline characteristics of breast cancer cases and matched controls.

Characteristics	Cases (*n* = 4875)	Controls (*n* = 48750)	*p*-Value ^g^
Age, ^a^ yrs, mean (SD) ^b^	61.31 (9.68)	61.26 (9.67)	
Females ^b^	4744 (97.3%)	47,440 (97.3%)	
Duration of follow-up, yrs, mean (SD) ^b^	5.16 (3.7)	5.15 (3.7)	
Socioeconomic status, *n* (%)			<0.001 ^h^
Low	1550 (33.7%)	18,974 (41.4%)	
Medium	1991 (43.3%)	18,344 (40.1%)	
High	1058 (23%)	8465 (18.5%)	
BMI, *n* (%)			0.428
<19	26 (0.6%)	333 (0.8%)	
19–24.5	669 (16.2%)	6895 (16.7%)	
24.6–30	1662 (40.3%)	16,289 (39.5%)	
≥30	1769 (42.9%)	17,725 (43%)	
Ethnicity			0.035 ^h^
Arabic	458 (9.39%)	7262 (14.9%)	
Haridi	117 (2.4%)	1293 (2.65%)	
Jewish non-Haridi	4223 (86.6%)	39,236 (80.5%)	
Others	1 (0.02%)	20 (4.1%)	
Missing	76 (1.55%)	939 (1.93%)	
Smoking status	1301 (26.7%)	12,331 (25.29%)	0.034 ^h^
Family history of Breast Cancer	643 (13.2%)	2631 (5.4%)	<0.01 ^h^
Comorbidities (%)			
Hyperlipidemia	4045 (83%)	40,457 (83%)	0.985
Ischemic heart disease	821 (16.8%)	8781 (18%)	0.042 ^h^
Heart failure	393 (8.1%)	4009 (8.4%)	0.433
Cardiovascular disease	1363 (28.0%)	14,771 (30.3%)	0.001 ^h^
Peripheral vascular disease	142 (2.9%)	1313 (2.7%)	0.368
Chronic kidney disease	425 (8.7%)	4296 (8.8%)	0.825
Chronic lung disease	687 (14.0.3%)	6944 (14.24%)	0.795
Comorbidity score, mean (SD)	1.75 (0.02)	1.78 (0.01)	0.165
Antihypertensive drugs (ever users), *n* (%)			
ACEIs and/or ARBs	3538 (72.6%)	35,094 (72%)	0.384
CCBs	1928 (39.5%)	19,400 (39.8%)	0.727
Diureticsc ^c^	2687 (55.11%)	27,024 (55.43%)	0.672
BBs	2725 (55.9%)	26,205 (53.8%)	0.047 ^h^
α-Blockersd ^d^	203 (4.2%)	1879 (3.9%)	0.255
α2 agonists ^e^	46 (0.9%)	555 (1.1%)	0.282
Hormone replacement therapy ^f^	390 (8%)	3245 (6.7%)	<0.001

ACEIs—angiotensin-converting enzyme inhibitors; ARBs—angiotensin receptor blockers; BBs—b-blockers; BMI—body mass index; CCBs—calcium channel blockers; COPD—chronic obstructive pulmonary disease; SD—standard deviation. ^a^ Age at index date. ^b^ Matching variables. ^c^ Furosemide, hydrochlorothiazide, amiloride hydrochloride with hydrochlorothiazide, spironolactone. ^d^ Prazosin, doxazosin. ^e^ Clonidine, Methyldopa. ^f^ Estradiol, Norethisterone, Estriol, Estrogen. ^g^ by two-sided ^χ^2 test. ^h^ statistically significant difference, *p*-value < 0.05. The differences are considered statistically insignificant with *p*-value > 0.05.

## Data Availability

The data presented in this study are available on request from the corresponding author. The data are not publicly available due to CHS restrictions.

## Supplementary Materials

The following supporting information can be downloaded at: https://www.mdpi.com/article/10.3390/cancers14092344/s1, Table S1: Antihypertensive Drugs categorized by Anatomical Therapeutic and Chemical Index; Table S2: Calcium Channel Blockers, categorized by Anatomical Therapeutic and Chemical Index; Table S3: Hormone Replacement Therapy categorized by Anatomical Therapeutic and Chemical Index; Table S4: Evaluation of potential confounders assessed by univariate conditional regression.

## References

[1] DeSantis C.E., Ma J., Gaudet M.M., Newman L.A., Miller K.D., Goding Sauer A., Jemal A., Siegel R.L. (2019). Breast cancer statistics, 2019. CA Cancer J. Clin..

[2] Coughlin S.S., Ekwueme D.U. (2009). Breast cancer as a global health concern. Cancer Epidemiol..

[3] The Israel Cancer Association—Breast Cancer [Internet]. https://en.cancer.org.il/.

[4] Pahor M., Guralnik J.M., Ferrucci L., Corti M.C., Salive M.E., Cerhan J.R., Wallace R.B., Havlik R.J. (1996). Calcium-channel blockade and incidence of cancer in aged populations. Lancet.

[5] Fitzpatrick A.L., Daling J.R., Furberg C.D., Kronmal R.A., Weissfeld J.L. (1997). Use of calcium channel blockers and breast carcinoma risk in postmenopausal women. Cancer.

[6] Li C.I., Daling J.R., Tang M.-T.C., Haugen K.L., Porter P.L., Malone K.E. (2013). Use of antihypertensive medications and breast cancer risk among women aged 55 to 74 years. JAMA Intern. Med..

[7] Rosenberg L., Rao R.S., Palmer J.R., Strom B.L., Stolley P.D., Zauber A.G., Warshauer M.E., Shapiro S. (1998). Calcium channel blockers and the risk of cancer. J. Am. Med. Assoc..

[8] Bergman G.J., Khan S., Danielsson B., Borg N. (2014). Breast cancer risk and use of calcium channel blockers using Swedish population registries. JAMA Intern. Med..

[9] Li C.I., Malone K.E., Weiss N.S., Boudreau D.M., Cushing-Haugen K.L., Daling J.R. (2003). Relation between use of antihypertensive medications and risk of breast carcinoma among women ages 65–79 years. Int. J. Am. Cancer Soc..

[10] Largent J.A., Bernstein L., Horn-Ross P.L., Marshall S.F., Neuhausen S., Reynolds P., Ursin G., Zell J.A., Ziogas A., Anton-Culver H. (2010). Hypertension, antihypertensive medication use, and breast cancer risk in the California teachers study cohort. Cancer Causes Control.

[11] Mason R.P. (1999). Calcium channel blockers, apoptosis and cancer: Is there a biologic relationship?. J. Am. Coll. Cardiol..

[12] Shih J.H., Kao L.T., Chung C.H., Liao G.S., Fann L.Y., Chien W.C., Li I.H. (2020). Protective association between calcium channel blocker use and breast cancer recurrence in postsurgical women: A population-based case-control study in Taiwan. J. Clin. Pharmacol..

[13] Sørensen H.T., Olsen J.H., Mellemkjær L., Thulstrup A.M., Steffensen F.H., McLaughlin J.K., Baron J.A. (2000). Cancer risk and mortality in users of calcium channel blockers: A cohort study. Cancer.

[14] Meier C.R., Derby L.E., Jick S.S., Jick H. (2000). Angiotensin-converting enzyme inhibitors, calcium channel blockers, and breast cancer. Arch. Intern. Med..

[15] Jick H., Jick S., Derby L.E., Vasilakis C., Myers M.W., Meier C.R. (1997). Calcium-channel blockers and risk of cancer. Lancet.

[16] Michels K.B., Rosner B.A., Walker A.M., Stampfer M.J., Manson J.E., Colditz G.A., Hennekens C.H., Willett W.C. (1998). Calcium channel blockers, cancer incidence, and cancer mortality in a cohort of U.S. Women: The nurses’ health study. Cancer.

[17] Fryzek J.P., Poulsen A.H., Lipworth L., Pedersen L., Nørgaard M., McLaughlin J.K., Friis S. (2006). A cohort study of antihypertensive medication use and breast cancer among Danish women. Breast Cancer Res. Treat..

[18] Devore E.E., Kim S., Ramin C.A., Wegrzyn L.R., Massa J., Holmes M.D., Michels K.B., Tamimi R.M., Forman J.P., Schernhammer E.S. (2015). Antihypertensive medication use and incident breast cancer in women. Breast Cancer Res. Treat..

[19] Wilson L.E., D’Aloisio A.A., Sandler D.P., Taylor J.A. (2016). Long-term use of calcium channel blocking drugs and breast cancer risk in a prospective cohort of US and Puerto Rican women. Breast Cancer Res..

[20] Brasky T.M., Krok-Schoen J.L., Liu J., Chlebowski R.T., Freudenheim J.L., Lavasani S., Margolis K.L., Qi L., Reding K.W., Shields P.G. (2017). Use of calcium channel blockers and breast cancer risk in the women’s health initiative. Cancer Epidemiol. Prev. Biomark..

[21] Raebel M.A., Zeng C., Cheetham T.C., Smith D.H., Feigelson H.S., Carroll N.M., Goddard K., Tavel H.M., Boudreau D.M., Shetterly S. (2017). Risk of breast cancer with long-term use of calcium channel blockers or angiotensin-converting enzyme inhibitors among older women. Am. J. Epidemiol..

[22] Azoulay L., Soldera S., Yin H., Bouganim N. (2016). Use of calcium channel blockers and risk of breast cancer: A population-based cohort study. Epidemiology.

[23] Chen Q., Zhang Q., Zhong F., Guo S., Jin Z., Shi W., Chen C., He J. (2014). Association between calcium channel blockers and breast cancer: A meta-analysis of observational studies. Pharmacoepidemiol. Drug Saf..

[24] Wright C.M., Moorin R.E., Chowdhury E.K., Stricker B.H., Reid C.M., Saunders C.M., Hughes J.D. (2017). Calcium channel blockers and breast cancer incidence: An updated systematic review and meta-analysis of the evidence. Cancer Epidemiol..

[25] Li W., Shi Q., Wang W., Liu J., Li Q., Hou F. (2014). Calcium channel blockers and risk of breast cancer: A meta-analysis of 17 observational studies. PLoS ONE.

[26] Thakur A.A., Wang X., Garcia-Betancourt M.M., Forse R.A. (2018). Calcium channel blockers and the incidence of breast and prostate cancer: A meta-analysis. J. Clin. Pharm. Ther..

[27] Copland E., Canoy D., Nazarzadeh M., Bidel Z., Ramakrishnan R., Woodward M., Chalmers J., Teo K.K., Pepine C.J., Davis B.R. (2021). Antihypertensive treatment and risk of cancer: An individual participant data meta-analysis. Lancet Oncol..

[28] Cohen R., Rabin H., Membership in Sick Funds, 2016 (2017). National Insurance Institute [Internet]. https://www.btl.gov.il/Publications/survey/Documents/seker289/seker_289.pdf.

[29] Clalit’s Database and the Research Institute—Mor Research Applications [Internet]. https://www.mor-research.com/.

[30] Structure and Principles WHO Collaborating Centre for Drug Statistics Methodology [Internet]. https://www.whocc.no/atc/structure_and_principles/.

[31] Central Bureau of Statistics of Israel Person reporting hypertension. 2009 [Internet]. https://www.cbs.gov.il/he/publications/doclib/2013/health_survey09_1500/pdf/t07.pdf.

[32] International Statistical Classification of Diseases and Related Health Problems Classification of Diseases (ICD) [Internet]. https://www.who.int/standards/classifications/classification-of-diseases.

[33] Leader A., Zelikson-Saporta R., Pereg D., Spectre G., Rozovski U., Raanani P., Hermoni D., Lishner M. (2017). The effect of combined aspirin and clopidogrel treatment on cancer incidence. Am. J. Med..

[34] Azrielant S., Tiosano S., Watad A., Mahroum N., Whitby A., Comaneshter D., Cohen A.D., Amital H. (2017). Correlation between systemic lupus erythematosus and malignancies: A cross-sectional population-based study. Immunol. Res..

[35] Dagan A., Segal G., Tiosano S., Watad A., Neumann S.G., Comaneshter D., Cohen A.D., Amital H. (2017). Coexistent malignant conditions in rheumatoid arthritis—A population-based cross-sectional study. Int. J. Clin. Pract..

[36] Strengthening the Reporting of Observational Studies in Epidemiology. https://www.strobe-statement.org/.

[37] Grassmann F., Yang H., Eriksson M., Azam S., Czene K. (2021). Mammographic features are associated with cardiometabolic disease risk and mortality. Eur. Heart J..

[38] Wu C.Y., Chang C.K., Robson D., Jackson R., Chen S.J., Hayes R.D., Stewart R. (2013). Evaluation of smoking status identification using electronic health records and open-text information in a large mental health case register. PLoS ONE.

