# Supplementary material for: Calcium Channel Blocker Use and the Risk for Breast Cancer: A Population-Based Nested Case-Control Study
Source: Cancers (Basel). 2022 May 9;14(9):2344. doi: 10.3390/cancers14092344 (PMC9101086; doi:10.3390/cancers14092344)
Supplement: Supplementary file 1 [file cancers-14-02344-s001.zip › cancers-1705420-supplementary.pdf]

Supplementary Materials: Calcium Channel Blocker Use and the Risk for Breast Cancer: A Population-Based Nested Case-Control Study

Victoria Rotshild, Bruria Hirsh Raccah, Muna Gazawe and Ilan Matok

Table S1. Antihypertensive Drugs categorized by Anatomical Therapeutic and Chemical Index [1].

Antihypertensive drugs	ATC4 Code
α-blockers	C02CA
Amiloride	C03DB01
Amiloride hydrochloride + Hydrochlorothiazide	C03EA
Angiotensin-converting enzyme inhibitors (ACEIs)	C09BB
Angiotensin receptor blockers (ARBs)	C09CA
Beta-blockers (BBs)	C07
Calcium Channel Blockers (CCB)	C08CA
Clonidine	C02AC
Furosemide	C03CA01
Methyldopa	C02AB
Spironolactone	C03DA01
Thiazide diuretics	C03A

Table S2. Calcium Channel Blockers, categorized by Anatomical Therapeutic and Chemical Index [1].

CCBs	ATC5 Code	DDD [1]
Non-Dihydropyridines		
Diltiazem	C08DB01	240 mg
Verapamil	C08DA01	240 mg
Dihydropyridines		
Amlodipine	C08CA01	5 mg
Felodipine	C08CA02	5 mg
Lercanidipine	C08CA13	10 mg
Nifedipine	C08CA05	30 mg

[1] DDD: Defined Daily Dose (DDD) codes.

Table S3. Hormone Replacement Therapy categorized by Anatomical Therapeutic and Chemical Index [1].

Hormone Replacement Therapy	ATC5 Code
Conjugated Estrogens	G03CA57
Dienogest and Estrogen	G03FA15
Drospirenone and Estrogen	G03FA17
Estradiol	G03CA03
Levonorgestrel and estrogen	G03FB09
Norethisterone and Estrogen	G03FA01
Norethisterone and Estrogen	G03FB05
Tibolone	G03CX01

Table S4. Evaluation of potential confounders assessed by univariate conditional regression.

Confounder	*p*-value	Odds Ratio	95% CI	
			Lower	Upper
Hormone Replacement Therapy	<0.001	1.232	1.102	1.377
Comorbidity Score	0.272	0.987	0.965	1.010
Smoking Status	0.030	1.078	1.007	1.154
Ethnicity				
Ethnicity- Arabic	0.788	1.318	0.177	9.843
Ethnicity- Haredi Jewish	0.532	1.902	0.253	14.303
Ethnicity –Other Jewish	0.422	2.276	0.305	16.966
Body Mass Index (BMI)				
BMI < 25	0.163	1.339	0.889	2.018
BMI 25–30	0.128	1.371	0.914	2.057
BMI > 30	0.117	1.384	0.922	2.077
Family history of Breast Cancer	<0.001	2.675	2.439	2.933
Socioeconomic Status				
Low Socioeconomic Status	<0.001	1.335	1.245	1.431
High Socioeconomic Status	<0.001	1.521	1.400	1.652
